# Differences in hospitalizations associated with severe COVID-19 disease among foreign- and Swedish-born

**DOI:** 10.1093/eurpub/ckad009

**Published:** 2023-02-07

**Authors:** Sol P Juárez, Agneta Cederström, Siddartha Aradhya, Mikael Rostila

**Affiliations:** Centre for Health Equity Studies (CHESS), Stockholm University/Karolinska Institutet, Stockholm, Sweden; Department of Public Health Sciences, Stockholm University, Stockholm, Sweden; Centre for Health Equity Studies (CHESS), Stockholm University/Karolinska Institutet, Stockholm, Sweden; Department of Public Health Sciences, Stockholm University, Stockholm, Sweden; Demography Unit (SUDA), Department of Sociology, Stockholm University, Stockholm, Sweden; Centre for Health Equity Studies (CHESS), Stockholm University/Karolinska Institutet, Stockholm, Sweden; Department of Public Health Sciences, Stockholm University, Stockholm, Sweden

## Abstract

**Background:**

Differences in pre-existing health conditions are hypothesized to explain immigrants’ excess COVID-19 mortality compared to natives. In this study, we evaluate whether immigrants residing in Sweden before the outbreak were more likely to be hospitalized for conditions associated with severe COVID-19 disease.

**Methods:**

A cohort study using population-register data was conducted with follow-up between 1 January 1997 and 31 December 2017. Poisson regression was fitted to estimate incidence rate ratio (RR) and 95% confident intervals (95% CI) for 10 causes of hospitalization.

**Results:**

Compared to Swedish-born individuals, most immigrant groups showed a decreased risk of hospitalization for respiratory chronic conditions, CVD, cancer, chronic liver conditions and neurological problems. All immigrant groups had increased risk of hospitalization for tuberculosis [RR between 88.49 (95% CI 77.21; 101.40) for the Horn of Africa and 1.69 (95% CI 1.11; 2.58) for North America], HIV [RR between 33.23 (95% CI 25.17; 43.88) for the rest of Africa and 1.31 (95% CI 0.93; 1.83) for the Middle East] and, with a few exceptions, also for chronic kidney conditions, diabetes and thalassemia.

**Conclusions:**

Foreign-born individuals—including origins with excess COVID-19 mortality in Sweden—did not show increased risk of hospitalizations for most causes associated with severe COVID-19 disease. However, all groups showed increased risks of hospitalization for tuberculosis and HIV and, with exceptions, for chronic kidney conditions, diabetes and thalassemia. Although studies should determine whether these health conditions explain the observed excess COVID-19 mortality, our study alerts to an increased risk of hospitalization that can be avoidable via treatment or preventive measures.

## Introduction

Immigrants and ethnic minorities have been excessively affected by the COVID-19 pandemic worldwide, showing higher rates of hospitalization and death compared to host populations.^1^ Sweden has not been an exception in this regard, despite its generous approach to immigrant integration and equal access to healthcare and social protection for all documented immigrants. Although with differences across regions of origin, most immigrant groups in Sweden have experienced excess risk of hospital admission for COVID-19 and related mortality^2–4^ compared to natives. These differences are partly explained by factors associated with greater exposure to SARS-CoV-2 infection, such as low income, occupations with high interpersonal contact or limited possibilities of working remotely, number of working-age household members and neighborhood population density.^3^^,^^4^

The greater burden experienced by immigrants and ethnic minorities^5^ across national contexts, after considering factors related to ‘differential exposure’ to the virus,^6^^,^^7^ has raised the question about the role of ‘differential effects’ of COVID-19 via susceptibility^7^^,^^8^—which is also referred to as vulnerability.^6^ Differential susceptibility has been posited to operate either through genetic^9^ or group-specific modifiable hazards, including vitamin D deficiency^10^ and pre-existing health conditions.^11^

To date, no peer-reviewed study has specifically examined the impact of underlying health conditions on the excess COVID-19 burden among immigrants in Sweden. There is currently a working paper^12^ and a report published by the National Board of Health and Welfare^13^ that finds little to no contribution of underlying conditions to migrants’ excess COVID-19 mortality and morbidity. However, neither of these studies covered the conditions that have been associated with severe COVID-19,^14^ and that are also overrepresented among some immigrant groups (e.g. tuberculosis and HIV). Furthermore, although there is extensive research on the health of immigrant populations, previous studies have predominantly focused on comparing overall mortality differences with natives.^15^ Studies on specific causes of death and hospitalizations are relatively scarce and do not necessarily cover the conditions associated with severe COVID-19.

The aim of this study is to evaluate the extent to which immigrant groups residing in Sweden before the onset of the COVID-19 outbreak were more likely to be hospitalized for health conditions that have been associated with severe COVID-19. It is particularly important to identify at-risk groups that can be targeted by public health measures (e.g. vaccination).

## Methods

### Study population and study design

A cohort study design was conducted using health and sociodemographic information from multiple population-based registers linked via pseudonymized personal identification numbers. Specifically, we identified the study population (including their country of birth and birth year) using the Total Population Register (1968).^16^ Using personal identification numbers,^17^ we linked information on inpatient care (1964) from the National Patient Register^18^ and sociodemographic information from the Longitudinal Integration Database for Health Insurance and Labour Market Studies (1990).^19^ The follow-up started in 1997, the year the individual turned 20, or first immigration, and continued until first cause-specific hospitalization (outcome), death, missing socioeconomic information, emigration or 31 December 2017, whichever occurred first. We censored where socioeconomic information was missing to ensure that the person-years are comparable across models. We excluded 9886 individuals due to missing information on the country of birth.

This study was approved by the Regional Ethical Review Board of Stockholm (decision no. 2017/716-31/5).

### Outcome

We studied 10 causes of hospitalizations associated with severe COVID-19 disease.^14^ This list includes: chronic kidney disease (ICD-10 code: N18), diabetes (E08–E13), cardiovascular diseases (CVD: E78, G45–G46, I10–I13, I20–I26, I63–I66, I80–I82), neurological problems (G20, G21, G30, G35–G37), chronic respiratory diseases (J40–J47), tuberculosis (A15–A19), HIV (B20–B24), chronic liver disease (K70–K77), cancer (C00–C96) and sickle cell disease or thalassemia (D56–D59).

### Region of birth

Region of birth was defined based on the country of birth and categorized into eight geographical groups: Finland, rest of Nordics, rest of EU28/EEA, non-EU28/EEA Europe, Middle East, Horn of Africa, rest of Africa, rest of Asia (excluding Middle East) and North and South America. We included Finland as a separate group given the large share of immigrants from this country in Sweden, and also since they display a distinctive health pattern from other Nordic immigrant groups with more adverse outcomes related to alcohol and other substance misuse.^20^^,^^21^

### Statistical analysis

We fitted Poisson regression with offset logarithm of follow-up time (person-time) in order to estimate incidence rate ratios (RRs) with 95% confidence intervals (95% CIs) for cause-specific hospitalization by region of origin. The rates were defined as the event divided by total-time-at-risk measured as time since entry (1997, year of 20th birthday, or first arrival) censoring subject at the time of hospitalization, missing socioeconomic information, emigration, death or end of follow-up (2017). The models examined the first time of hospitalization for each of the 10 causes studied independently from each other. Calendar year and age (categorized as 20–40, 41–50, 51–60, 61–70, 71–80, 81–90, 91–100 and >101) were used to define the time-scale. Two model specifications were considered. Model 1, adjusted for sex, was fitted to estimate the direct effect of the region of origin on cause-specific hospitalization using the Swedish-born population as reference category. Model 2 is an extension of Model 1 including socioeconomic factors measured at the start of follow-up and each age span. This includes family type (defined as: couples with children, couples without children, single with children and single), education (primary, secondary, post-secondary and unknown) and individual disposable income (in quintiles) i.e. including salary or any benefit (parental leave, sick leave or social allowance). Model 2 examines the extent to which the differences attenuate when considering socioeconomic characteristics in the residing country. We conducted sex- and age-stratified analyses.

All analyses were performed using R version 4.1.1.

## Results

Although with variation by region of origin, compared to natives, most immigrant groups were younger on average and overrepresented in the categories of partners with children, lower education and in lower income levels (Supplementary table S1 for detailed descriptive information).

**Figure 1:**
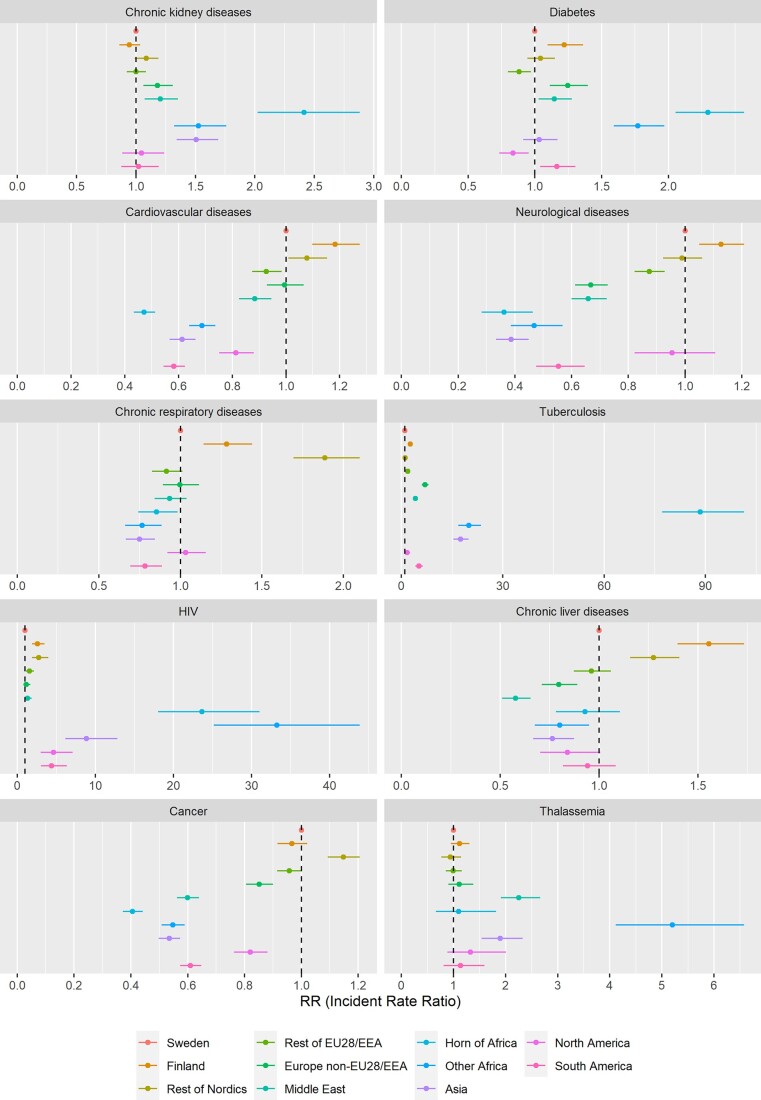
Incidence RRs with 95% CIs for specific cause of hospitalization by foreign-born group of origin compared to the Swedish-born individuals. Adjusted for age, sex and calendar year

Figure 1 presents the results from the Poisson regression models for the 10 specific causes of hospitalization by region of birth and sex, with Swedish-born being the reference group (Supplementary table S2 for number of events and Supplementary table S3 for estimates). Despite there being variation across regions and causes of hospitalization, the results display some general patterns. Compared to Swedish-born individuals, most immigrant groups experienced increased risk of hospitalization for tuberculosis [RR ranging from 88.49 (95% CI 77.21; 101.40) for the Horn of Africa to 1.69 (95% CI 1.11; 2.58) for North America] and HIV [with RR between 33.23 (95% CI 25.17; 43.88) for the rest of Africa and 1.15 (95% CI 0.77; 1.70) for non-EU28/EEA Europe]. The estimates were attenuated but point in the same direction after the inclusion of socioeconomic controls.

Relative to Swedish-born individuals, immigrants from Finland and other Nordic countries showed increased risk of hospitalization for chronic liver conditions [RR 1.56 (95% CI 1.40; 1.73) and 1.28 (95% CI 1.16; 1.41), respectively], chronic respiratory diseases [RR 1.28 (95% CI 1.14; 1.44) and 1.89 (95% CI 1.69; 2.10)] and CVD [RR 1.18 (95% CI 1.10; 1.27) and 1.08 (95% CI 1.01; 1.15)]. In addition, immigrants from Finland showed an increased risk of hospitalizations for neurological problems [RR 1.13 (95% CI 1.05; 1.21)] while immigrants from other Nordic countries for cancer [RR 1.15 (95% CI 1.09; 1.21)]. The inclusion of socioeconomic controls did not attenuate the strength of the association, except for other Nordics in relation to CVD [RR 1.00 (95% CI 0.94; 1.07)]. In contrast, the rest of immigrant groups showed either no difference or a decreased risk of hospitalization for the above-mentioned causes of hospitalization.

Larger variation across immigrant groups was observed for diabetes, chronic kidney diseases and thalassemia when compared to the Swedish-born individuals. With the exception of immigrants from North America, rest of EU28/EEA Europe, rest of Asia and the rest of Nordics, for whom there was either no difference or decreased risks, all other origins showed an increased risk of hospitalization for diabetes with RR ranging from 2.30 (95% CI 2.05; 2.57) for the Horn of Africa to 1.14 (95% CI 1.03; 1.28) for the Middle East. For the latter group, the association changed direction with the inclusion of socioeconomic controls. As for chronic kidney diseases, there was a clear divide between Europe (rest of EU28/EEA, Finland and rest of Nordics) and the Americas (both North and South) vs. the rest of the world. Thus, while Europe and the Americas showed no differences in chronic kidney diseases, the rest of the world displayed an increased risk [RR ranging from 2.42 (95% CI 2.02; 2.88) for the Horn of Africa and 1.18 (95% CI 1.06; 1.31) for non-EU28/EEA]. Finally, three groups showed a clear increased risk of hospitalization for thalassemia: immigrants from the rest of Africa (RR 5.20 95% CI 4.11; 6.58), the Middle East (RR 2.25 95% CI 1.91; 2.66) and rest of Asia (RR 1.89 95% CI 1.54; 2.33). These estimates were attenuated but pointed in the same direction after the inclusion of socioeconomic controls.

Sex-specific analyses showed overall consistent patterns (Supplementary table S4) with most differences driven by the magnitude of the effect sizes. Changes in directionality were observed among immigrants from the Middle East and non-EU28/EEA Europe in relation to chronic kidney conditions, from the Middle East in relation to diabetes and from Finland in relation to neurological problems, for whom women showed an increased risk. No difference or decreased risk was observed among men. The opposite is shown among immigrants from the rest of EU28/EEA Europe in relation to HIV, for whom an increased risk was observed among men (in line with the general results) but not among women. Sub-analyses also indicated that the lack of effects shown among immigrants from the rest of the Nordics (in relation to tuberculosis) and South America (for chronic liver conditions) were driven by men since women showed increased risk for these causes of hospitalization. Finally, the lack of effect observed among immigrants from South America in relation to chronic kidney diseases was most likely attributed to mixed findings between men and women, where men showed a decreased risk and women an increased risk. The opposite was true for immigrants from Middle East for HIV, for whom the lack of effect was driven by the lower risk among women.

Age-stratified analyses (above and below 65 years of age) showed overall consistent results (i.e. same directionality) across age groups (Supplementary table S5). The only exception was observed among immigrants from Asia and South America in relation to chronic liver conditions, where the lower risks compared to Swedes only held for immigrants below age 65.

## Discussion

### Summary of the results

Our study shows large variability across causes of hospitalization and regions of origins, but a few patterns are worth highlighting. First, immigrants did not show a consistently increased risk of hospitalizations for all the causes associated with severe COVID-19 disease. In fact, most immigrant groups (including those that experienced excess COVID-19 mortality) showed decreased risks of hospitalization for many causes, including respiratory chronic conditions, CVD, cancer, chronic liver conditions and neurological problems. Finns were the only group that stood out showing increased risk of hospitalization for all conditions except for chronic kidney disease and cancer. Second, despite large heterogeneity across origins in relation to chronic kidney disease, diabetes and thalassemia (with both lower and higher risks compared to Swedish-born), most immigrant groups showed increased risks of hospitalization for tuberculosis and HIV.

The health pattern observed in our study suggests that some immigrant groups were more likely to face a double burden of disease —having higher risks of suffering from both infectious diseases (specifically HIV and Tuberculosis) and ‘man-made’ diseases (such as chronic kidney and liver diseases or diabetes). This pattern, however, is not restricted to immigrants from low-income countries, where infectious diseases play a greater role, as immigrants from Finland and non-EU28/EEA European countries also displayed similar patterns. Further studies should confirm this ‘double disease burden’ and examine whether this reflects large variability within immigrant groups or whether this is indicative of some groups being affected by co-morbidity.

### Relation to previous and future research

Our study shows different risks of hospitalizations between immigrants and Swedes. Future studies should evaluate the underlying causes behind these findings by focusing on the role of social determinants and their intermediate factors (underlying risk behaviors, including physical inactivity and smoking, for example). The excess risk of hospitalization for chronic kidney disease warrants special attention since this health condition has been particularly associated (via allostatic overload)^22^^,^^23^ with stressful life events, socioeconomic disadvantages, discrimination and racism, all of which disproportionately affect non-European migrants in Sweden.^24–26^ Psychosocial stress has also been shown to induce insulin resistance leading the development of diabetes,^27^ i.e. another cause of hospitalization for which most immigrants in our study showed higher risks. It is important to highlight that the increased risk of hospitalization for diabetes in immigrants from low-income countries attenuated after the inclusion of socioeconomic controls, except for African immigrants. This finding may not be entirely surprising considering that our controls do not necessarily take into consideration ethnic/racial discrimination, for example. Yet, it is also worth mentioning that immigrants from Finland (which are less like to affected by ethnic/racial discrimination) also experienced excess risk for diabetes that is not explained by socioeconomic factors. As such, a careful consideration of the causes behind the higher risk is needed.

In our study women for most origins show similar (if not higher) risks of hospitalization as compared to men for most diseases. Therefore, our sex-specific findings do not support the ‘double health advantage’ observed among immigrant women (i.e. a decreased risk of mortality compared to their male counterparts who, in turn, show lower risks than Swedish natives).^28^

Specifically in relation to COVID-19 research, our study shows some suggestive patterns worth investigating in future research. The immigrant groups who experienced pronounced excess COVID-19 mortality since the beginning of the pandemic in Stockholm (i.e. from the Middle East and Africa)^2^^,^^3^ show higher risks for some morbidities associated with severe COVID-19 disease, such as tuberculosis, HIV (only significant for Africa), diabetes, chronic kidney conditions and thalassemia (not significant for the Horn of Africa). Although this health pattern is not exclusive for these origins (in fact most immigrants are affected by these and other causes), the risk levels (especially for tuberculosis and HIV) tend to be higher for individuals born in Middle East, Africa and Asia. Future studies should examine the contribution of co- and multi-morbidity in the excess COVID-19 mortality among immigrants with particular attention to tuberculosis. In addition to a recent systematic review and meta-analysis that shows an increase in COVID-19 severity among individuals with ‘co-occurrence’ of tuberculosis,^29^ a rapid systematic review of the literature published in 2020 concluded that people with ‘pre‐existing’ tuberculosis had a higher risk of suffering from serious complications from COVID‐19.^30^ Moreover, concern exists as to whether COVID-19 may ‘activate’ latent tuberculosis,^31^ as also observed in historical epidemics (like the 1918 ‘Spanish flu’^32^^,^^33^ and the 2009 Influenza A -H1N1^34^), leading to more severe COVID-19 disease and a possible increase of the incidence of tuberculosis post-pandemic.^31^

Our findings suggest that none of the underlying heath conditions considered in previous Swedish studies^12^^,^^13^ that evaluated the excess risk of COVID-19 mortality and morbidity were particularly relevant for immigrants. However, it is also worth mentioning that the causes of hospitalization for which immigrants have shown a higher risk in this study (such as tuberculosis, HIV and thalassemia) are unlikely to fully explain inequalities in COVID-19 mortality due to their low prevalence. In fact, in light of the existing empirical evidence,^11^ it is questionable that susceptibility *per se* can fully explain excess COVID-19 mortality. At the same time, it is reasonable to expect that tuberculosis and HIV (which already suggest a poor management of the disease) indicate higher vulnerability on average for some immigrant groups and for some individuals within certain immigrant groups.

In this study, Finns showed increased risks of hospitalization for almost all conditions. This finding is consistent with previous studies showing poor health outcomes for this group^15^^,^^21^ and suggests that susceptibility could play a key role in explaining their excess COVID-19 burden in Sweden. With this said, the fact that most immigrant groups (and specifically those excessively affected by the pandemic) show lower risks for many causes of hospitalization suggests that accounting for these conditions in a model might only contribute to exacerbate the differences in COVID-19 outcomes compared to Swedes.

Researchers in Sweden have hypothesized that vitamin D deficiency could be behind the increased risk of COVID-19 mortality shown among immigrants from Somalia.^35^ International research on this association remains inconclusive due to the lack of relevant confounders and evaluation of large samples.^36^ Our data does not allow us to draw any conclusions about the role of vitamin D in explaining excess mortality among immigrants. However, it is relevant to highlight that none of the immigrant groups (except from Finland and other Nordics) in this study showed an increased risk of hospitalization for respiratory chronic conditions, which has been argued to be affected by vitamin D deficiency. In fact, the group of immigrants from the Horn of Africa shows the lowest risk among all groups after the inclusion of socioeconomic controls.

### Public health implications

Besides the contribution of our findings to COVID-19 research, our study also has direct public health implications. Given that most of the selected causes of hospitalization are avoidable (either with timely treatment or with preventive public health measures), our findings could indicate a poor capacity to detect problems in an early phase. This is particularly the case for tuberculosis, which nowadays is considered a preventable and treated disease, and for which most groups showed an excess risk of hospitalization.

The generalization of these findings and their implications should be done with caution since hospitalization is strongly dependent on healthcare access, treatment and preventive measures, which vary across contexts.

### Strengths and limitations

The major strength of our study is the use of high-quality, longitudinal, individual-level data that covers health and social information for all individuals registered in Sweden. This allows us to assess the causes of hospitalizations associated with severe COVID-19 between immigrants and Swedes before the start of the pandemic with and without considering socioeconomic characteristics.

The major limitation of this study is the lack of individual-level information on COVID-19 mortality linked to prior hospitalization data. This means that we cannot determine whether immigrants who were in emergency care or died from COVID-19 had previously experienced any of the health conditions examined in this study. Such examination would require the availability of COVID-19 outcomes and also the consideration of milder health conditions, obesity and health risk behaviors. Although the severity of the outcome (hospitalization) may compromise the examination of susceptibility, since a portion of the population might be affected by chronic problems, information on hospitalization is relevant. First, it is less likely to suffer from problems of under-diagnosis, which are common when using less severe outcomes, especially among immigrants. Second, the lack of early diagnosis or the poor management of the disease (as suspected from preventable causes of hospitalization) may put individuals in a more difficult situation to fight against COVID-19 disease. Those who are aware of being at higher risk might take actions to protect themselves against infection, as suggested by a Norwegian study.^37^

The 10 causes of hospitalizations examined in this study have been those reported in high-quality studies to be correlated with severe COVID-19.^14^ However, since our knowledge about COVID-19 is developing fast, we do not rule out the possibility that other relevant conditions may be omitted.

Adjustments for socioeconomic characteristics should also be considered with caution since the quality of the information depends on the migratory status. Education in the registers is self-reported for immigrants who have not completed their education in Sweden and there is a substantial proportion of missing data. Similarly, disposable income may not be comparable across groups over time. For example, newly arrived immigrants (including those highly educated) who arrive as refugees might be entirely dependent on welfare benefits during the first years. In any case, this limitation influences our ability to explain some of the observed differences but should not compromise the overall findings. In line with this, this study also lacks relevant social factors available in registers (like occupation) and other not so directly measured (like discrimination and structural and interpersonal racism).

In conclusion, immigrants did not show increased risk of hospitalizations for most causes associated with severe COVID-19. However, all immigrant origins showed increased risks of hospitalization for tuberculosis and HIV and, with exceptions, also for chronic kidney conditions, diabetes and thalassemia. Studies should examine the extent to which the above-mentioned causes of hospitalization contribute to the excess COVID-19 mortality among immigrants in Sweden. Nonetheless, understanding the underlying health differentials between Swedish and foreign groups of different origins can help prevent these avoidable causes of hospitalization (via vaccination programs, treatment or preventive measures) and hopefully also reduce native-immigrant inequalities due to differential vulnerability in future pandemics.

## Supplementary Material

ckad009_Supplementary_Data

## Data Availability

The data are available from Statistics Sweden and the National Board of Health and Welfare (Socialstyrelsen) under license for the current study, and are not publicly available.
