# Supplementary material for: Differences in hospitalizations associated with severe COVID-19 disease among foreign- and Swedish-born
Source: Eur J Public Health. 2023 Feb 7;33(3):522–7. doi: 10.1093/eurpub/ckad009 (PMC10234654; doi:10.1093/eurpub/ckad009)
Supplement: ckad009_Supplementary_Data [file ckad009_supplementary_data.docx]

**Supplementary material**

**Table S1. Characteristics of the study population**

|  | **Sweden** | | **Finland** | | **Rest of Nordic** | | **Rest of EU28/EEA** | | **Europe non-EU28/EEA** | |
| --- | --- | --- | --- | --- | --- | --- | --- | --- | --- | --- |
|  | **N=7785872 (79.8%)** | | **N=215779 (2.2%)** | | **N=149004 (1.5%)** | | **419056 (4.3%)** | | **N=222397 (2.3%)** | |
|  | **(50.4% women)** | | **(58.8% w)** | | **(50.0% w)** | | **(48.0% w)** | | **(52.3% w)** | |
|  | **Person years** | **%** | **Person years** | **%** | **Person years** | **%** | **Person years** | **%** | **Person years** | **%** |
| **Age** |  |  |  |  |  |  |  |  |  |  |
| 20-39 | 38059877.49 | 33.0 | 359459.9478 | 11.5 | 327850.0553 | 22.7 | 1377507.83 | 34.2 | 1191258.934 | 42.1 |
| 40-49 | 19565787.05 | 17.0 | 528876.1684 | 16.9 | 224952.3888 | 15.6 | 718773.5535 | 17.9 | 624251.8248 | 22.1 |
| 50-59 | 19573317.16 | 17.0 | 774647.1673 | 24.7 | 242045.1151 | 16.8 | 674582.9283 | 16.8 | 469120.9856 | 16.6 |
| 60-69 | 16942486.78 | 14.7 | 779750.5656 | 24.9 | 254246.0623 | 17.6 | 594895.0765 | 14.8 | 303934.6565 | 10.8 |
| 70-79 | 12297057.84 | 10.7 | 494618.1736 | 15.8 | 238857.4357 | 16.6 | 427173.8702 | 10.6 | 174705.591 | 6.2 |
| 80-89 | 7321455.524 | 6.3 | 173408.4192 | 5.5 | 134034.1209 | 9.3 | 195213.9788 | 4.9 | 55749.22977 | 2.0 |
| 90-99 | 1530124.259 | 1.3 | 20423.19097 | 0.7 | 20403.42457 | 1.4 | 34238.32252 | 0.9 | 7297.519302 | 0.3 |
| 100+ | 33841.05448 | 0.0 | 308.6371663 | 0.0 | 340.5082136 | 0.0 | 1345.121492 | 0.0 | 514.3430527 | 0.0 |
| **Disposable Income (Quintiles)** |  |  |  |  |  |  |  |  |  |  |
| 1st lowest | 19051814.1 | 16.5 | 353683.7026 | 11.3 | 411397.8696 | 28.5 | 1398694.186 | 34.8 | 977612.6836 | 34.6 |
| 2nd | 23334855.73 | 20.2 | 665183.3125 | 21.2 | 277984.0232 | 19.3 | 706592.8279 | 17.6 | 571335.0126 | 20.2 |
| 3rd | 24228244.2 | 21.0 | 744753.0136 | 23.8 | 271289.5267 | 18.8 | 683026.0635 | 17.0 | 484224.3133 | 17.1 |
| 4th | 24522794.93 | 21.3 | 763826.2077 | 24.4 | 232717.2606 | 16.1 | 602999.5926 | 15.0 | 442623.0936 | 15.7 |
| 5th hightest | 24046063.53 | 20.9 | 591005.8355 | 18.9 | 237289.6119 | 16.4 | 595671.8642 | 14.8 | 219812.707 | 7.8 |
| **Family Type** |  |  |  |  |  |  |  |  |  |  |
| Partner with children | 40311003.05 | 35.0 | 746658.0368 | 23.8 | 339098.324 | 23.5 | 1137607.232 | 28.3 | 1216896.388 | 43.0 |
| Partner without children | 25121125.15 | 21.8 | 909153.2787 | 29.0 | 398466.4767 | 27.6 | 866133.9306 | 21.5 | 511772.5491 | 18.1 |
| Single with children | 10357864.88 | 9.0 | 250224.1767 | 8.0 | 88414.07755 | 6.1 | 304225.3084 | 7.6 | 240471.9635 | 8.5 |
| Single/Other | 39393923.11 | 34.2 | 1212416.58 | 38.7 | 604699.4137 | 41.9 | 1679022.063 | 41.7 | 726466.9093 | 25.7 |
| **Education attainment** |  |  |  |  |  |  |  |  |  |  |
| Basic | 28949944.33 | 25.1 | 1149125.977 | 36.7 | 399843.3467 | 27.7 | 630097.1224 | 15.7 | 612523.4401 | 21.7 |
| Secondary | 58559564.88 | 50.8 | 1317571.01 | 42.1 | 518285.5862 | 35.9 | 1419104.063 | 35.3 | 1130074.707 | 40.0 |
| Post-secondary | 26486082.09 | 23.0 | 552387.9987 | 17.6 | 296227.5959 | 20.5 | 1204942.247 | 29.9 | 561044.8345 | 19.8 |
| *Unknown* | 1188324.887 | 1.0 | 99367.08606 | 3.2 | 216321.7632 | 15.0 | 732845.1019 | 18.2 | 391964.8285 | 13.9 |

*Continues..*

|  | **Middle East** | | **Horn of Africa** | | **Other Africa** | | **Rest of Asia** | | **North America** | | **South America** | |
| --- | --- | --- | --- | --- | --- | --- | --- | --- | --- | --- | --- | --- |
|  | **N=392062 (4.0%)** | | **N=96500 (1%)** | | **N=78750 (0.8%)** | | **N=274814 (2.8%)** | | **N=52472(0.5%)** | | **N=74881 (0.8%)** | |
|  | **(44.4% women)** | | **(48.7% w)** | | **(42.4% w)** | | **(54.0% w)** | | **(47.5% w)** | | **(51.6% w)** | |
|  | **Person years** | **%** | **Person years** | **%** | **Person years** | **%** | **Person years** | **%** | **Person years** | **%** | **Person years** | **%** |
| **Age** |  |  |  |  |  |  |  |  |  |  |  |  |
| 20-39 | 2006705.006 | 51.2 | 493842.4963 | 64.1 | 397509.8244 | 55.9 | 1448941.236 | 62.7 | 204970.446 | 46.2 | 457267.1 | 49.4 |
| 40-49 | 956742.9443 | 24.4 | 168030.6413 | 21.8 | 173316.7202 | 24.4 | 479094.1039 | 20.7 | 96948.36042 | 21.9 | 202874.2 | 21.9 |
| 50-59 | 568846.1756 | 14.5 | 67727.8332 | 8.8 | 88704.96728 | 12.5 | 232047.0241 | 10.0 | 57298.99617 | 12.9 | 149276.2 | 16.1 |
| 60-69 | 232226.6329 | 5.9 | 26273.70168 | 3.4 | 36024.65448 | 5.1 | 94309.41793 | 4.1 | 31246.13854 | 7.0 | 74522.46 | 8.0 |
| 70-79 | 108624.0965 | 2.8 | 11071.6117 | 1.4 | 12000.02108 | 1.7 | 39210.37748 | 1.7 | 29205.88508 | 6.6 | 28705.59 | 3.1 |
| 80-89 | 38957.69678 | 1.0 | 2667.986516 | 0.3 | 3417.892745 | 0.5 | 13888.03908 | 0.6 | 19658.83244 | 4.4 | 11191.09 | 1.2 |
| 90-99 | 6028.464819 | 0.2 | 257.7610541 | 0.0 | 490.7464066 | 0.1 | 2087.087817 | 0.1 | 3885.986653 | 0.9 | 2233.584 | 0.2 |
| 100+ | 654.3085558 | 0.0 | 10.20506502 | 0.0 | 27.03983573 | 0.0 | 110.3061602 | 0.0 | 155.8958932 | 0.0 | 222.1218 | 0.0 |
| **Disposable Income (Quintiles)** |  |  |  |  |  |  |  |  |  |  |  |  |
| 1st lowest | 1755463.003 | 44.8 | 347972.9177 | 45.2 | 334958.2464 | 47.1 | 1232810.198 | 53.4 | 196403.2122 | 44.3 | 342664 | 37.0 |
| 2nd | 748996.2054 | 19.1 | 125397.1414 | 16.3 | 101396.1198 | 14.3 | 333019.4144 | 14.4 | 63903.98807 | 14.4 | 172697.3 | 18.6 |
| 3rd | 525159.1566 | 13.4 | 86933.08344 | 11.3 | 91388.80729 | 12.8 | 271049.4137 | 11.7 | 55051.93005 | 12.4 | 154036.8 | 16.6 |
| 4th | 400368.1795 | 10.2 | 80384.63073 | 10.4 | 84203.38761 | 11.8 | 217968.8146 | 9.4 | 53967.50071 | 12.2 | 150288.2 | 16.2 |
| 5th hightest | 245980.2209 | 6.3 | 51066.03566 | 6.6 | 58473.76927 | 8.2 | 143525.5449 | 6.2 | 65379.27125 | 14.7 | 87876.66 | 9.5 |
| **Family Type** |  |  |  |  |  |  |  |  |  |  |  |  |
| Partner with children | 1809390.962 | 46.2 | 202453.0192 | 26.3 | 261606.6122 | 36.8 | 845217.704 | 36.6 | 136925.3449 | 30.9 | 348948.1 | 37.7 |
| Partner without children | 537484.6233 | 13.7 | 63010.78227 | 8.2 | 105914.0563 | 14.9 | 307994.1493 | 13.3 | 79914.27616 | 18.0 | 87139.41 | 9.4 |
| Single with children | 322427.4005 | 8.2 | 111485.4146 | 14.5 | 73608.11745 | 10.3 | 214598.2025 | 9.3 | 37203.31465 | 8.4 | 158234.7 | 17.1 |
| Single/Other | 1006663.78 | 25.7 | 314804.5928 | 40.9 | 229291.5443 | 32.2 | 830563.3296 | 36.0 | 180662.9665 | 40.7 | 313240.7 | 33.8 |
| **Education attainment** |  |  |  |  |  |  |  |  |  |  |  |  |
| Basic | 1028580.094 | 26.2 | 215008.8766 | 27.9 | 133891.1021 | 18.8 | 480604.6544 | 20.8 | 58008.40308 | 13.1 | 203621.8 | 22.0 |
| Secondary | 1124871.876 | 28.7 | 216788.4584 | 28.2 | 206616.9362 | 29.0 | 627090.5522 | 27.2 | 112179.5753 | 25.3 | 391605.2 | 42.3 |
| Post-secondary | 809926.734 | 20.7 | 81674.37912 | 10.6 | 188176.3224 | 26.4 | 556253.106 | 24.1 | 177105.0318 | 39.9 | 217548.7 | 23.5 |
| *Unknown* | 712588.061 | 18.2 | 178282.0948 | 23.2 | 141735.9696 | 19.9 | 534425.0729 | 23.1 | 87412.89211 | 19.7 | 94787.25 | 10.2 |

**Table S2. Number of events by region of origin and cause of hospitalization**

| **Region of origin** | **N** | **%** | **Chronic Kidney Disease** | **Diabetes** | **Cardiovascular Disease** | | **Neurological problems** | | **Chronic Respiratory Disease** | | **Tuberculosis** | | **HIV** | | **Chronic Liver Disease** | | **Cancer** | | **Sickle cell /Thelassia** | |
| --- | --- | --- | --- | --- | --- | --- | --- | --- | --- | --- | --- | --- | --- | --- | --- | --- | --- | --- | --- | --- |
| Sweden | 7785872 | 79.8 | 88883 | 239925 | 2673835 | 111178 | | 376482 | | 3969 | | 1639 | | 69252 | | 1836158 | | 7364 | |  |
| Finland | 215779 | 2.2 | 2980 | 7918 | 112423 | 3624 | | 16413 | | 301 | | 102 | | 3750 | | 63420 | | 179 | |  |
| Rest of Nordics | 149004 | 1.5 | 1394 | 3127 | 48196 | 1695 | | 12522 | | 62 | | 51 | | 1241 | | 32630 | | 118 | |  |
| Rest of EU28/EEA | 419056 | 4.3 | 3127 | 5634 | 86130 | 2981 | | 10679 | | 244 | | 106 | | 2439 | | 58212 | | 182 | |  |
| Europe non-EU28/EEA | 222397 | 2.3 | 2459 | 4278 | 45873 | 1602 | | 6243 | | 605 | | 38 | | 1216 | | 30698 | | 69 | |  |
| Middle East | 392062 | 4.0 | 2905 | 4823 | 44656 | 1833 | | 5421 | | 497 | | 74 | | 1231 | | 23466 | | 1243 | |  |
| Horn of Africa | 96500 | 1.0 | 1163 | 1627 | 3008 | 119 | | 640 | | 2539 | | 403 | | 358 | | 2882 | | 17 | |  |
| Rest of Africa | 78750 | 0.8 | 622 | 1137 | 4941 | 180 | | 599 | | 456 | | 582 | | 336 | | 3563 | | 867 | |  |
| Rest of Asia | 274814 | 2.8 | 1628 | 1989 | 12635 | 525 | | 1790 | | 1218 | | 387 | | 657 | | 9847 | | 553 | |  |
| North America | 52472 | 0.5 | 247 | 486 | 6043 | 268 | | 891 | | 37 | | 29 | | 239 | | 4276 | | 40 | |  |
| South America | 74881 | 0.8 | 708 | 1026 | 6066 | 317 | | 947 | | 147 | | 61 | | 414 | | 5884 | | 25 | |  |
|  |  |  | **17233** | **32045** | **369971** | **13144** | | **56145** | | **6106** | | **1833** | | **11881** | | **234878** | | **3293** | |  |

**Table S3. Results from Poisson regression models. Incidence Risk Ratios (RR) and 95% Confidence Intervals for cause-specific hospitalizations by region of birth. Minimally (age) and adjusted models (age, education, income and family type) are presented**

|  | Chronic Kidney Disease | | Diabetes | | Cardiovascular Diseases | | Neurological Problems | |
| --- | --- | --- | --- | --- | --- | --- | --- | --- |
| **Region of origin** | Age adjusted | Age + SES adjusted | Age adjusted | Age + SES adjusted | Age adjusted | Age + SES adjusted | Age adjusted | Age + SES adjusted |
| Sweden | 1 (ref) | 1 (ref) | 1 (ref) | 1 (ref) | 1 (ref) | 1 (ref) | 1 (ref) | 1 (ref) |
| Finland | 0.94  (0.86-1.03) | 0.87  (0.80-0.94) | 1.22  (1.09-1.36) | 1.08  (1.01-1.15) | 1.18  (1.10-1.27) | 1.12  (1.05-1.18) | 1.13  (1.05-1.21) | 1.11  (1.03-1.19) |
| Rest of Nordics | 1.08  (0.99-1.19) | 1.03  (0.95-1.12) | 1.04  (0.94-1.15) | 0.91  (0.84-0.99) | 1.08  (1.01-1.15) | 1.00  (0.94-1.07) | 0.99  (0.92-1.06) | 0.98  (0.91-1.05) |
| Rest of EU28/EEA | 1.00  (0.92-1.08) | 1.00  (0.93-1.07) | 0.88  (0.80-0.97) | 0.82  (0.75-0.89) | 0.93  (0.87-0.98) | 0.91  (0.86-0.96) | 0.87  (0.82-0.93) | 0.88  (0.83-0.94) |
| Europe non-EU28/EEA | 1.18  (1.06-1.31) | 1.08  (1.00-1.18) | 1.25  (1.11-1.40) | 1.04  (0.96-1.13) | 1.00  (0.93-1.07) | 0.89  (0.83-0.94) | 0.67  (0.61-0.73) | 0.66  (0.60-0.72) |
| Middle East | 1.20  (1.07-1.35) | 1.08  (0.98-1.20) | 1.14  (1.03-1.28) | 0.88  (0.81-0.96) | 0.88  (0.83-0.95) | 0.75  (0.70-0.81) | 0.66  (0.60-0.72) | 0.66  (0.59-0.72) |
| Horn of Africa | 2.42  (2.02-2.88) | 1.99  (1.68-2.35) | 2.30  (2.05-2.57) | 1.56  (1.39-1.75) | 0.47  (0.43-0.51) | 0.38  (0.34-0.41) | 0.36  (0.28-0.46) | 0.35  (0.27-0.45) |
| Rest of Africa | 1.52  (1.32-1.76) | 1.42  (1.24-1.62) | 1.77  (1.59-1.97) | 1.44  (1.30-1.60) | 0.69  (0.64-0.74) | 0.63  (0.58-0.68) | 0.47  (0.38-0.57) | 0.46  (0.38-0.56) |
| Asia | 1.51  (1.34-1.69) | 1.37  (1.22-1.54) | 1.03  (0.91-1.17) | 0.81  (0.69-0.94) | 0.61  (0.57-0.66) | 0.53  (0.48-0.58) | 0.39  (0.33-0.45) | 0.38  (0.32-0.44) |
| North America | 1.05  (0.88-1.24) | 1.07  (0.90-1.26) | 0.84  (0.73-0.95) | 0.79  (0.69-0.90) | 0.81  (0.75-0.88) | 0.80  (0.74-0.87) | 0.95  (0.82-1.11) | 0.95  (0.82-1.11) |
| South America | 1.02  (0.88-1.19) | 0.96  (0.83-1.10) | 1.16  (1.04-1.30) | 0.99  (0.91-1.08) | 0.58  (0.54-0.62) | 0.54  (0.51-0.58) | 0.55  (0.48-0.65) | 0.55  (0.48-0.65) |

Continued...

|  | Chronic Liver Disease | | Cancer | | Thalassemia | |
| --- | --- | --- | --- | --- | --- | --- |
| **Region of origin** | Age adjusted | Age + SES adjusted | Age adjusted | Age + SES adjusted | Age adjusted | Age + SES adjusted |
| Sweden | 1 (ref) | 1 (ref) | 1 (ref) | 1 (ref) | 1 (ref) | 1 (ref) |
| Finland | 1.56  (1.40-1.73) | 1.38  (1.29-1.49) | 0.97  (0.92-1.02) | 0.94  (0.89-0.99) | 1.11  (0.95-1.31) | 1.05  (0.90-1.23) |
| Rest of Nordics | 1.28  (1.16-1.41) | 1.17  (1.09-1.26) | 1.15  (1.09-1.21) | 1.13  (1.08-1.18) | 0.94  (0.77-1.15) | 0.88  (0.72-1.07) |
| Rest of EU28/EEA | 0.96  (0.87-1.06) | 0.93  (0.86-1.00) | 0.96  (0.91-1.00) | 0.98  (0.94-1.02) | 0.99  (0.85-1.16) | 0.96  (0.82-1.12) |
| Europe non-EU28/EEA | 0.80  (0.71-0.89) | 0.74  (0.68-0.81) | 0.85  (0.81-0.90) | 0.84  (0.80-0.88) | 1.11  (0.90-1.38) | 1.00  (0.81-1.23) |
| Middle East | 0.58  (0.51-0.65) | 0.52  (0.48-0.57) | 0.60  (0.56-0.64) | 0.59  (0.56-0.63) | 2.25  (1.91-2.66) | 1.95  (1.63-2.32) |
| Horn of Africa | 0.93  (0.78-1.11) | 0.73  (0.62-0.87) | 0.41  (0.37-0.44) | 0.39  (0.35-0.42) | 1.10  (0.66-1.82) | 0.87  (0.52-1.46) |
| Rest of Africa | 0.80  (0.67-0.95) | 0.72  (0.62-0.83) | 0.55  (0.51-0.59) | 0.55  (0.51-0.59) | 5.20  (4.11-6.58) | 4.63  (3.63-5.90) |
| Asia | 0.76  (0.67-0.87) | 0.68  (0.60-0.77) | 0.53  (0.50-0.57) | 0.52  (0.48-0.56) | 1.89  (1.54-2.33) | 1.63  (1.32-2.02) |
| North America | 0.84  (0.70-1.01) | 0.83  (0.71-0.98) | 0.82  (0.76-0.88) | 0.84  (0.78-0.90) | 1.33  (0.88-2.00) | 1.29  (0.85-1.94) |
| South America | 0.94  (0.82-1.08) | 0.86  (0.76-0.97) | 0.61  (0.57-0.65) | 0.62  (0.59-0.65) | 1.14  (0.81-1.60) | 1.05  (0.75-1.48) |

Continued...

|  | Chronic Respiratory Disease | | Tuberculosis | | HIV | |
| --- | --- | --- | --- | --- | --- | --- |
| **Region of origin** | Age adjusted | Age + SES adjusted | Age adjusted | Age + SES adjusted | Age adjusted | Age + SES adjusted |
| Sweden | 1 (ref) | 1 (ref) | 1 (ref) | 1 (ref) | 1 (ref) | 1 (ref) |
| Finland | 1.28  (1.14-1.44) | 1.13  (1.05-1.22) | 2.66  (2.31-3.06) | 2.41  (2.12-2.74) | 2.57  (1.88-3.51) | 2.03  (1.56-2.65) |
| Rest of Nordics | 1.89  (1.69-2.10) | 1.71  (1.58-1.84) | 1.15  (0.89-1.50) | 0.99  (0.75-1.29) | 2.75  (1.90-3.97) | 2.01  (1.36-2.96) |
| Rest of EU28/EEA | 0.92  (0.83-1.01) | 0.90  (0.84-0.96) | 1.89  (1.64-2.18) | 1.65  (1.42-1.92) | 1.55  (1.12-2.14) | 1.14  (0.80-1.64) |
| North America | 1.00  (0.89-1.11) | 0.83  (0.77-0.89) | 6.96  (6.01-8.07) | 5.60  (4.92-6.38) | 1.15  (0.77-1.70) | 1.06  (0.73-1.54) |
| Europe non-EU28/EEA | 0.93  (0.84-1.04) | 0.75  (0.70-0.81) | 4.11  (3.56-4.75) | 3.00  (2.63-3.42) | 1.31  (0.93-1.83) | 1.07  (0.77-1.48) |
| Middle East | 0.85  (0.74-0.98) | 0.59  (0.52-0.67) | 88.49  (77.21-101.40) | 59.58  (52.69-67.37) | 23.66  (18.04-31.03) | 14.33  (10.68-19.22) |
| Horn of Africa | 0.77  (0.66-0.88) | 0.66  (0.58-0.75) | 19.93  (16.88-23.52) | 15.09  (12.79-17.79) | 33.23  (25.17-43.88) | 24.63  (18.71-32.41) |
| Rest of Africa | 0.75  (0.66-0.84) | 0.62  (0.56-0.70) | 17.46  (15.37-19.83) | 12.63  (11.10-14.38) | 8.88  (6.14-12.84) | 6.26  (4.40-8.89) |
| Rest of Asia | 1.03  (0.92-1.16) | 1.05  (0.95-1.17) | 1.69  (1.11-2.58) | 1.43  (0.94-2.18) | 4.62  (3.01-7.11) | 3.44  (2.18-5.43) |
| South America | 0.78  (0.69-0.89) | 0.68  (0.62-0.75) | 5.16  (4.18-6.36) | 4.36  (3.57-5.34) | 4.39  (3.03-6.37) | 3.54  (2.52-4.97) |

SES = education, income and family type

**Table S4. Results from Poisson regression models. Incidence Risk Ratios (RR) and 95% Confidence Intervals for cause-specific hospitalizations by region of birth by sex (adjusted for age)**

|  | Chronic Kidney Disease | | Diabetes | | Cardiovascular Diseases | | Neurological Problems | |
| --- | --- | --- | --- | --- | --- | --- | --- | --- |
| **Region of origin** | Women | Men | Women | Men | Women | Men | Women | Men |
| Sweden | 1(ref) |  | 1(ref) |  | 1(ref) |  | 1(ref) |  |
| Finland | 0.96  (0.83-1.10) | 0.94  (0.83-1.06) | 1.15  (0.98-1.35) | 1.30  (1.13-1.49) | 1.16  (1.04-1.30) | 1.21  (1.11-1.33) | 1.17  (1.07-1.28) | 1.05  (0.95-1.16) |
| Rest of Nordics | 1.02  (0.87-1.19) | 1.13  (1.01-1.27) | 1.07  (0.90-1.25) | 1.02  (0.91-1.15) | 1.10  (0.99-1.23) | 1.06  (0.98-1.13) | 0.93  (0.84-1.02) | 1.07  (0.98-1.17) |
| Rest of EU28/EEA | 1.00  (0.88-1.13) | 1.00  (0.90-1.11) | 0.91  (0.78-1.07) | 0.86  (0.76-0.96) | 0.93  (0.84-1.02) | 0.93  (0.87-0.99) | 0.89  (0.82-0.97) | 0.85  (0.78-0.93) |
| North America | 1.45  (1.24-1.69) | 1.03  (0.90-1.17) | 1.36  (1.13-1.64) | 1.16  (1.02-1.31) | 0.96  (0.85-1.08) | 1.03  (0.95-1.11) | 0.70  (0.62-0.79) | 0.63  (0.56-0.71) |
| Europe non-EU28/EEA | 1.72  (1.46-2.02) | 0.97  (0.85-1.11) | 1.30  (1.08-1.57) | 1.05  (0.93-1.19) | 0.79  (0.69-0.89) | 0.95  (0.88-1.03) | 0.76  (0.67-0.86) | 0.57  (0.50-0.64) |
| Middle East | 2.80  (1.97-3.98) | 2.23  (1.88-2.64) | 2.09  (1.76-2.49) | 2.43  (2.11-2.79) | 0.40  (0.35-0.47) | 0.52  (0.47-0.57) | 0.30  (0.21-0.43) | 0.42  (0.30-0.59) |
| Horn of Africa | 1.76  (1.34-2.31) | 1.45  (1.23-1.70) | 1.59  (1.32-1.91) | 1.85  (1.63-2.10) | 0.62  (0.55-0.70) | 0.72  (0.66-0.78) | 0.42  (0.30-0.58) | 0.50  (0.40-0.63) |
| Rest of Africa | 1.72  (1.46-2.03) | 1.36  (1.17-1.57) | 1.01  (0.85-1.21) | 1.06  (0.89-1.25) | 0.50  (0.45-0.57) | 0.73  (0.66-0.80) | 0.32  (0.27-0.39) | 0.48  (0.38-0.60) |
| Rest of Asia | 1.10  (0.85-1.43) | 1.01  (0.82-1.26) | 0.80  (0.65-0.99) | 0.86  (0.72-1.02) | 0.86  (0.76-0.98) | 0.77  (0.70-0.85) | 0.98  (0.80-1.19) | 0.93  (0.74-1.16) |
| South America | 1.36  (1.09-1.70) | 0.82  (0.68-0.98) | 1.15  (0.96-1.37) | 1.18  (1.02-1.35) | 0.58  (0.52-0.64) | 0.59  (0.54-0.64) | 0.57  (0.47-0.70) | 0.53  (0.42-0.67) |

Continued…

|  | Chronic Liver Diseases | | Cancer | | Thalassemia | |
| --- | --- | --- | --- | --- | --- | --- |
| **Region of origin** | Women | Men | Women | Men | Women | Men |
| Sweden | 1(ref) |  | 1(ref) |  | 1(ref) |  |
| Finland | 1.24  (1.06-1.44) | 1.36  (1.16-1.60) | 0.97  (0.90-1.05) | 0.96  (0.90-1.03) | 1.17  (0.96-1.42) | 1.03  (0.79-1.35) |
| Rest of Nordics | 1.88  (1.62-2.19) | 1.89  (1.64-2.19) | 1.14  (1.06-1.22) | 1.16  (1.08-1.24) | 0.91  (0.72-1.16) | 0.97  (0.68-1.38) |
| Rest of EU28/EEA | 0.90  (0.78-1.03) | 0.94  (0.82-1.08) | 0.95  (0.90-1.02) | 0.96  (0.90-1.03) | 0.93  (0.76-1.15) | 1.07  (0.86-1.35) |
| North America | 0.91  (0.78-1.06) | 1.11  (0.95-1.30) | 0.84  (0.78-0.90) | 0.87  (0.80-0.94) | 1.14  (0.86-1.52) | 1.08  (0.79-1.48) |
| Europe non-EU28/EEA | 0.92  (0.80-1.05) | 0.95  (0.81-1.11) | 0.68  (0.63-0.74) | 0.53  (0.49-0.58) | 2.80  (2.24-3.50) | 1.73  (1.37-2.18) |
| Middle East | 0.79  (0.65-0.96) | 0.92  (0.76-1.12) | 0.47  (0.42-0.53) | 0.35  (0.31-0.39) | 1.67  (0.96-2.90) | 0.50  (0.15-1.65) |
| Horn of Africa | 0.64  (0.52-0.80) | 0.85  (0.70-1.02) | 0.58  (0.52-0.64) | 0.53  (0.48-0.58) | 6.73  (4.92-9.22) | 4.08  (2.89-5.76) |
| Rest of Africa | 0.73  (0.62-0.85) | 0.78  (0.65-0.92) | 0.59  (0.55-0.64) | 0.46  (0.41-0.51) | 2.25  (1.77-2.86) | 1.37  (0.96-1.96) |
| Rest of Asia | 1.05  (0.90-1.21) | 1.01  (0.85-1.21) | 0.82  (0.75-0.90) | 0.82  (0.73-0.91) | 1.61  (0.95-2.71) | 1.02  (0.52-2.00) |
| South America | 0.84  (0.71-0.98) | 0.71  (0.59-0.85) | 0.64  (0.59-0.69) | 0.58  (0.53-0.63) | 1.15  (0.74-1.76) | 1.13  (0.65-1.95) |

Continued…

|  | Chronic Respiratory Disease | | Tuberculosis | | HIV | |
| --- | --- | --- | --- | --- | --- | --- |
| Region of Origin | Women | Men | Women | Men | Women | Men |
| Sweden | 1(ref) |  | 1(ref) |  | 1(ref) |  |
| Finland | 1.24  (1.06-1.44) | 1.36  (1.16-1.60) | 2.28  (1.89-2.76) | 3.19  (2.62-3.88) | 2.01  (1.18-3.43) | 2.95  (2.02-4.31) |
| Rest of Nordics | 1.88  (1.62-2.19) | 1.89  (1.64-2.19) | 1.44  (1.01-2.05) | 0.88  (0.61-1.27) | 2.28  (1.06-4.88) | 2.92  (1.90-4.50) |
| Rest of EU28/EEA | 0.90  (0.78-1.03) | 0.94  (0.82-1.08) | 1.82  (1.47-2.25) | 1.96  (1.62-2.38) | 0.94  (0.49-1.82) | 1.75  (1.19-2.56) |
| North America | 0.91  (0.78-1.06) | 1.11  (0.95-1.30) | 8.23  (6.62-10.23) | 5.89  (4.91-7.08) | 1.36  (0.69-2.67) | 1.11  (0.69-1.79) |
| Europe non-EU28/EEA | 0.92  (0.80-1.05) | 0.95  (0.81-1.11) | 4.70  (3.80-5.82) | 3.69  (3.04-4.47) | 0.54  (0.23-1.26) | 1.48  (1.00-2.18) |
| Middle East | 0.79  (0.65-0.96) | 0.92  (0.76-1.12) | 103.21  (85.89-124.02) | 77.47  (63.35-94.73) | 54.42  (39.23-75.50) | 15.16  (10.82-21.25) |
| Horn of Africa | 0.64  (0.52-0.80) | 0.85  (0.70-1.02) | 22.54  (18.04-28.16) | 18.27  (14.51-23.00) | 113.40  (83.78-153.49) | 14.71  (10.64-20.33) |
| Rest of Africa | 0.73  (0.62-0.85) | 0.78  (0.65-0.92) | 19.02  (15.95-22.68) | 16.10  (13.50-19.20) | 24.86  (16.59-37.27) | 2.83  (1.94-4.11) |
| Rest of Asia | 1.05  (0.90-1.21) | 1.01  (0.85-1.21) | 2.03  (1.10-3.75) | 1.42  (0.80-2.53) | 1.92  (0.52-7.13) | 5.29  (3.33-8.42) |
| South America | 0.84  (0.71-0.98) | 0.71  (0.59-0.85) | 5.95  (4.43-7.98) | 4.50  (3.35-6.04) | 4.76  (2.54-8.90) | 4.37  (2.77-6.90) |

**Table S5. Results from Poisson regression models. Incidence Risk Ratios (RR) and 95% Confidence Intervals for cause-specific hospitalizations by region of birth by age (and adjusted by age)**

|  | Chronic Kidney Disease | | Diabetes | | Cardiovascular Diseases | | Neurological Problems | |
| --- | --- | --- | --- | --- | --- | --- | --- | --- |
| **Region of origin** | < 65 | >65 | < 65 | >65 | < 65 | >65 | < 65 | >65 |
| Sweden | 1(ref) |  | 1(ref) |  | 1(ref) |  | 1(ref) |  |
| Finland | 0.97  (0.86-1.09) | 0.77  (0.68-0.87) | 1.23  (1.10-1.38) | 1.07  (0.95-1.21) | 1.26  (1.19-1.33) | 1.01  (0.95-1.07) | 1.05  (0.96-1.15) | 1.09  (1.01-1.17) |
| Rest of Nordics | 0.77  (0.65-0.90) | 1.01  (0.87-1.16) | 0.89  (0.78-1.01) | 0.87  (0.77-0.98) | 0.96  (0.88-1.04) | 0.91  (0.85-0.97) | 0.84  (0.73-0.97) | 0.85  (0.77-0.93) |
| Rest of EU28/EEA | 0.73  (0.64-0.84) | 1.06  (0.95-1.19) | 0.65  (0.58-0.74) | 0.90  (0.80-1.02) | 0.76  (0.71-0.83) | 0.90  (0.85-0.96) | 0.66  (0.59-0.73) | 0.89  (0.81-0.97) |
| North America | 1.27  (1.11-1.45) | 0.95  (0.82-1.10) | 1.16  (1.01-1.32) | 1.27  (1.07-1.50) | 1.04  (0.97-1.10) | 0.90  (0.84-0.96) | 0.68  (0.61-0.77) | 0.65  (0.54-0.77) |
| Europe non-EU28/EEA | 1.07  (0.93-1.22) | 1.78  (1.53-2.08) | 1.00  (0.89-1.12) | 1.49  (1.25-1.76) | 0.88  (0.82-0.95) | 0.83  (0.75-0.90) | 0.70  (0.62-0.80) | 0.53  (0.42-0.67) |
| Middle East | 2.10  (1.74-2.53) | 2.16 (1.31-3.57) | 2.17  (1.92-2.46) | 1.10  (0.89-1.37) | 0.43  (0.38-0.48) | 0.30  (0.24-0.38) | 0.35  (0.24-0.49) | 0.14  (0.07-0.28) |
| Horn of Africa | 1.30  (1.06-1.60) | 2.62  (1.90-3.61) | 1.57  (1.39-1.78) | 1.72  (1.30-2.27) | 0.68  (0.62-0.75) | 0.57  (0.48-0.68) | 0.43  (0.32-0.58) | 0.53  (0.29-0.96) |
| Rest of Africa | 1.31  (1.13-1.52) | 1.73  (1.36-2.19) | 0.87  (0.75-1.01) | 1.39  (1.12-1.72) | 0.56  (0.50-0.62) | 0.63  (0.58-0.69) | 0.30  (0.24-0.38) | 0.50  (0.35-0.72) |
| Rest of Asia | 0.77  (0.57-1.04) | 1.05  (0.79-1.40) | 0.66  (0.54-0.81) | 0.74  (0.60-0.91) | 0.50  (0.45-0.56) | 0.92  (0.85-1.00) | 0.78  (0.59-1.03) | 0.84  (0.65-1.08) |
| South America | 1.02  (0.84-1.24) | 1.15  (0.78-1.68) | 1.08  (0.95-1.23) | 1.30  (1.03-1.63) | 0.62  (0.57-0.67) | 0.57  (0.51-0.65) | 0.49  (0.38-0.62) | 0.81  (0.57-1.16) |

Continued…

|  | Chronic Liver Diseases | | Cancer | | Thalassemia | |
| --- | --- | --- | --- | --- | --- | --- |
| **Region of origin** | < 65 | >65 | < 65 | >65 | < 65 | >65 |
| Sweden | 1(ref) |  | 1(ref) |  | 1(ref) |  |
| Finland | 1.49  (1.32-1.68) | 1.07  (0.93-1.22) | 0.94  (0.90-0.98) | 0.83  (0.79-0.87) | 1.16  (0.93-1.45) | 0.87  (0.61-1.24) |
| Rest of Nordics | 1.12  (0.98-1.28) | 1.10  (0.94-1.28) | 0.96  (0.89-1.03) | 1.03  (0.96-1.09) | 0.69  (0.42-1.14) | 0.93  (0.67-1.29) |
| Rest of EU28/EEA | 0.72  (0.63-0.82) | 1.10  (0.97-1.25) | 0.77  (0.72-0.82) | 0.94  (0.89-1.00) | 0.82  (0.62-1.08) | 1.01  (0.74-1.38) |
| North America | 0.73  (0.64-0.82) | 1.00  (0.81-1.23) | 0.85  (0.80-0.89) | 0.79  (0.74-0.84) | 0.87  (0.61-1.24) | 1.40  (0.92-2.12) |
| Europe non-EU28/EEA | 0.53  (0.46-0.61) | 1.01  (0.78-1.31) | 0.59  (0.55-0.63) | 0.58  (0.53-0.63) | 2.85  (2.32-3.50) | 1.63  (1.02-2.59) |
| Middle East | 0.93  (0.75-1.16) | 0.99  (0.56-1.76) | 0.37  (0.33-0.42) | 0.34  (0.27-0.42) | 1.18  (0.64-2.19) | 0.67  (0.10-4.59) |
| Horn of Africa | 0.74  (0.60-0.91) | 0.44  (0.13-1.49) | 0.51  (0.46-0.57) | 0.65  (0.53-0.80) | 6.52  (4.86-8.74) | 3.38  (1.00-11.45) |
| Rest of Africa | 0.64  (0.54-0.76) | 1.92  (1.40-2.64) | 0.47  (0.43-0.52) | 0.59  (0.51-0.68) | 1.73  (1.32-2.27) | 3.08  (1.81-5.22) |
| Rest of Asia | 0.70  (0.55-0.90) | 0.79 (  0.51-1.22) | 0.58  (0.51-0.64) | 0.93  (0.85-1.01) | 1.10  (0.53-2.31) | 1.27  (0.60-2.70) |
| South America | 0.91  (0.76-1.09) | 1.81  (1.21-2.70) | 0.60  (0.56-0.65) | 0.72  (0.63-0.82) | 1.19  (0.71-1.99) | 1.12  (0.41-3.01) |

Continued…

|  | Chronic Respiratory Disease | | Tuberculosis | | HIV | |
| --- | --- | --- | --- | --- | --- | --- |
| Region of Origin | < 65 | >65 | < 65 | >65 | < 65 | >65 |
| Sweden | 1(ref) |  | 1(ref) |  | 1(ref) |  |
| Finland | 1.26  (1.10-1.44) | 1.20  (1.06-1.35) | 2.72  (2.20-3.35) | 2.19  (1.84-2.61) | 2.36  (1.61-3.45) | 1.17  (0.16-8.83) |
| Rest of Nordics | 1.57 (  1.36-1.82) | 1.63  (1.40-1.89) | 0.77  (0.45-1.30) | 1.15  (0.79-1.67) | 2.28  (1.43-3.64) | 1.39  (0.19-10.10) |
| Rest of EU28/EEA | 0.66  (0.58-0.76) | 0.99  (0.87-1.12) | 2.00  (1.63-2.44) | 1.46  (1.09-1.96) | 1.39  (0.95-2.03) | 1.00  (0.14-7.14) |
| North America | 0.90  (0.80-1.02) | 1.11  (0.98-1.25) | 7.41  (6.23-8.82) | 5.88  (4.50-7.68) | 0.95  (0.58-1.56) | 0.00  (0.00-0.00) |
| Europe non-EU28/EEA | 0.92  (0.81-1.03) | 1.04  (0.91-1.20) | 4.67  (3.88-5.63) | 3.46  (2.34-5.12) | 1.14  (0.75-1.73) | 4.11  (0.57-29.80) |
| Middle East | 0.92  (0.77-1.10) | 0.49  (0.37-0.64) | 115.65  (100.47-133.13) | 28.77  (18.70-44.27) | 25.79  (19.40-34.28) | 92.00  (31.26-270.78) |
| Horn of Africa | 0.87 (  0.72-1.04) | 0.60  (0.40-0.89) | 26.20  (21.97-31.24) | 5.24  (2.01-13.65) | 35.36  (26.25-47.63) | 92.50  (26.10-327.79) |
| Rest of Africa | 0.70  (0.60-0.82) | 0.82  (0.69-0.98) | 20.02  (17.03-23.53) | 10.98  (7.28-16.58) | 9.81  (6.82-14.10) | 0.00  (0.00-0.00) |
| Rest of Asia | 0.72  (0.58-0.89) | 1.08  (0.91-1.27) | 2.18  (1.23-3.87) | 1.23  (0.52-2.94) | 4.10  (2.44-6.87) | 0.00  (0.00-0.00) |
| South America | 0.89  (0.76-1.03) | 0.81  (0.64-1.03) | 6.32  (4.82-8.29) | 4.08  (2.09-7.96) | 4.76 (  3.15-7.19) | 0.00  (0.00-0.00) |
